# External validation of and improvement upon a model for the prediction of placenta accreta spectrum severity using prospectively collected multicenter ultrasound data

**DOI:** 10.1111/aogs.14941

**Published:** 2024-08-20

**Authors:** Magdalena Kolak, Stephen Gerry, Hubert Huras, Ammar Al Naimi, Karin A. Fox, Thorsten Braun, Vedran Stefanovic, Heleen van Beekhuizen, Olivier Morel, Alexander Paping, Charline Bertholdt, Pavel Calda, Zdenek Lastuvka, Andrzej Jaworowski, Egle Savukyne, Sally Collins, IS‐PAS group, Kinga M. Chalubinski, Katarzyna Kawka‐Paciorkowska, Mina Mhallem Gziri, Maddalena Morlando, Frederic Chantraine, Gita Strindfors, Anja Bluth, Petra Hanulikova, Ozhan M. Turan, Andrew Rubenstein, Albaro Jose Nieto‐Calvache

**Affiliations:** ^1^ Department of Obstetrics and Perinatology, Medical College Jagiellonian University Krakow Poland; ^2^ Centre for Statistics in Medicine University of Oxford Oxford UK; ^3^ Division of Obstetrics & Maternal Fetal Medicine, Department of Obstetrics and Gynecology University Hospital Frankfurt Goethe‐University Frankfurt Germany; ^4^ Department of Obstetrics and Gynecology Buergerhospital Frankfurt Germany; ^5^ Division of Maternal‐Fetal Medicine, Department of Obstetrics and Gynecology Baylor College of Medicine Houston Texas USA; ^6^ Department of Obstetrics Charité – Universitätsmedizin Berlin, Freie Universität Berlin and Humboldt‐Universität zu Berlin Berlin Germany; ^7^ Department of Obstetrics and Gynecology, Fetomaternal Medical Center Helsinki University Hospital and University of Helsinki Berlin Finland; ^8^ Department of Gynecological Oncology Erasmus MC Cancer Center Rotterdam The Netherlands; ^9^ Department of Obstetrics and Gynecology, CHRU‐ Nancy Université de Lorraine Nancy France; ^10^ IADI, INSERM Université de Lorraine Nancy France; ^11^ Department of Gynecology, Obstetrics and Neonatology, First Faculty of Medicine Charles University and General University Hospital in Prague Prague Czech Republic; ^12^ Hospital of Lithuanian University of Health Sciences Kaunas Clinics Lithuanian University of Health Sciences Kaunas Lithuania; ^13^ Nuffield Department of Women's and Reproductive Health University of Oxford Oxford UK

**Keywords:** abnormal adherent placenta, abnormal invasive placenta, placenta accreta spectrum, prediction model, probability

## Abstract

**Introduction:**

This study aimed to validate the Sargent risk stratification algorithm for the prediction of placenta accreta spectrum (PAS) severity using data collected from multiple centers and using the multicenter data to improve the model.

**Material and Methods:**

We conducted a multicenter analysis using data collected for the IS‐PAS database. The Sargent model's effectiveness in distinguishing between abnormally adherent placenta (FIGO grade 1) and abnormally invasive placenta (FIGO grades 2 and 3) was evaluated. A new model was developed using multicenter data from the IS‐PAS database.

**Results:**

The database included 315 cases of suspected PAS, of which 226 had fully documented standardized ultrasound signs. The final diagnosis was normal placentation in 5, abnormally adherent placenta/FIGO grade 1 in 43, and abnormally invasive placenta/FIGO grades 2 and 3 in 178. The external validation of the Sargent model revealed moderate predictive accuracy in a multicenter setting (*C*‐index 0.68), compared to its higher accuracy in a single‐center context (*C*‐index 0.90). The newly developed model achieved a *C*‐index of 0.74.

**Conclusions:**

The study underscores the difficulty in developing universally applicable PAS prediction models. While models like that of Sargent et al. show promise, their reproducibility varies across settings, likely due to the interpretation of the ultrasound signs. The findings support the need for updating the current ultrasound descriptors and for the development of any new predictive models to use data collected by different operators in multiple clinical settings.

## Graphical abstract

This study evaluated the Sargent risk stratification algorithm for predicting placenta accreta spectrum (PAS) severity across multiple centers using the IS‐PAS database. The algorithm's ability to differentiate between abnormally adherent (FIGO grade 1) and invasive placenta (FIGO grades 2 and 3) was assessed, and a new model was developed based on this multicenter data. Analysis of 315 suspected PAS cases revealed that the Sargent model had moderate predictive accuracy in a multicenter context (*C*‐index 0.68), lower than in a single‐center setting (*C*‐index 0.90). The new model achieved a *C*‐index of 0.74. The study highlights challenges in creating universally applicable PAS models, suggesting the need for updated ultrasound descriptors and new models incorporating data from diverse clinical environments.

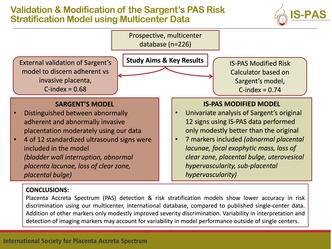

**Abbreviations**
AAP: abnormally adherent placenta
AIP: abnormal invasive placenta
CS: cesarean section
FIGO: International Federation of Gynecology and Obstetrics
PAS: placenta accreta spectrum
US: ultrasound

**Key message**

Models for prediction of PAS such as that proposed by Sargent et al. show promise in specific settings; however, their accuracy diminishes when applied to data collected in other centers. This highlights the need to improve the existing ultrasound descriptors.

## 1 INTRODUCTION

Placenta accreta spectrum (PAS) is a growing problem facing modern obstetrics. The incidence of PAS has increased over the last century from 1 in 30 000 pregnancies in the 1950s to 1 in 272 pregnancies in recent years.^1^, ^2^ PAS represents a severe obstetric complication that is characterized by abnormal attachment of the placenta into the uterine wall and often extending beyond the typical muscular margins. It can cause life‐threatening maternal hemorrhage and significantly affect maternal and neonatal morbidity and mortality.^3^ PAS is often managed by performing a hysterectomy with the placenta in situ, but other types of management are continuously evolving.^4^ Antenatal PAS prediction is an integral part of management that is constantly improving with the refinement of imaging techniques, for both ultrasound (US) and magnetic resonance imaging (MRI). Various multifactorial calculators for the prediction of PAS based on risk factors and imaging findings have been proposed over the past few years with most models developed from single‐center datasets and only discriminating between the presence and absence of PAS.^5^, ^6^, ^7^, ^8^, ^9^, ^10^ The earliest, in 2015, was the Placenta Accreta Index, which was a combination of clinical and US features. The score from 0 to 9 points gave the probability of invasion from 5% to 96%.^6^ Then, in 2016, Maymon's simple website calculator was published. In the presence or absence of three features (anterior location of placenta, placenta lacunae, demarcation), the probability of PAS was calculated from 1% to 87%.^7^ Prediction calculators created after 2020 also used MRI features or machine learning models.^5^, ^11^ These models have yet to demonstrate improved patient outcomes despite the increasing awareness of the condition.

In 2022, a model was proposed by Sargent et al. aimed at not only diagnosing PAS but also predicting its severity. It used the standardized sonographic descriptors developed by the European Working Group on Abnormally Invasive Placenta^12^ to differentiate between “adherent and invasive” PAS and non‐PAS cases with high accuracy, according to the FIGO clinical classification.^12^, ^13^, ^14^, ^15^

Until now, this model has not been externally validated; therefore, its utility in the prediction of PAS outside of the center in which it was developed was unknown. The main aim of this study was to externally validate the performance of the Sargent prediction model in a large international multicenter prospective dataset. Secondarily, we aimed to develop a modified version based on the multicenter database and to compare the performance of both models.

## 2 MATERIAL AND METHODS

The development of the model to predict the presence and severity of PAS based on the standardized US markers was fully described in Sargent et al.^12^ Briefly, theirs was a prospective, single‐center cohort study including cases at high risk of PAS. PAS diagnosis was made intraoperatively according to the FIGO clinical classification and confirmed by histopathological examination, resulting in three groups: normal placentation, FIGO grade 1 analogous to the abnormally adherent placenta (AAP: accreta), and FIGO grades 2 and 3 analogous to abnormally invasive placenta (AIP: increta or percreta). Each US sign was quantified with a *C*‐index; the final model was created using four variables, and these were placental lacunae, loss of clear zone, myometrial thinning, and bladder wall interruption. The *C*‐index measures the discrimination, which is the probability that the model will correctly identify a patient with a specific outcome from one without the outcome among two randomly selected patients (e.g., they had/or did not have PAS, AAP, or AIP). A *C*‐index value of 1 represents full discrimination (ability to classify correctly), and a value of 0.5 corresponds to random classification (i.e., the result is due to chance, the inability of the model to classify). A simple flow diagram was presented to calculate the predicted risk.^12^

Our external validation was performed using the International Society for Placenta Accreta Spectrum (IS‐PAS) database of suspected and confirmed PAS cases collected between January 1, 2020 to June 30, 2022 by 23 centers from 16 countries using a standardized, secured and password‐protected online data collection platform (FetView 2.0, Zeitgeist Health SE) (Editorial of this IS‐PAS Supplement). The database used the FIGO clinical classification and histopathology to diagnose the presence and severity of PAS.^15^ The calculations were performed on those cases with prenatal suspicion of PAS and fully documented standardized US descriptors. Due to the nature of the database and the paucity of cases with normal placentation, we examined the model's discrimination between AAP and AIP. FIGO grade 1 patients were classified as AAP, whereas grades 2 and 3 were classified as AIP. Several protocols were implemented to ensure the consistency of US data across different centers in the study. First, all the sonographers who participated in the study were specialists trained in evaluating PAS. Second, all centers perform US according to FIGO guidelines and EW‐AIP consensus. Moreover, to minimize variability in data interpretation, doubtful cases were discussed among centers.

Throughout the study, we adhered to the Transparent Reporting of a multivariable prediction model for Individual Prognosis or Diagnosis (TRIPOD) guidelines.^16^ This ensured the integrity and clarity of our research methodology and findings.^16^

### 2.1 Risk estimation and used sonographic signs

PAS risk estimation was taken from the original model development paper.^12^ The model used four out of 12 standardized US markers as dichotomous variables. These were the loss of clear zone, abnormal placenta lacunae, placental bulge, and bladder wall interruption. The model calculated the probabilities of normal placentation, AAP, and AIP using the following formulas:

LP1 = −2.22 + 2.14 (Loss of clear zone) + 1.71 (Abnormal placental lacunae) + 0.44 (Bladder wall interruption) + 3.03 (Placental bulge).

LP2 = −2.22 – 3.71 + 2.14 (Loss of clear zone) + 1.71 (Abnormal placental lacunae) + (2.96–0.44) (Bladder wall interruption) + 3.03 (Placental bulge).

The probabilities were calculated as follows:

- Probability of Normal Placentation: Prob(Normal) = 1–eLP1/1 + eLP1.
- Probability of AAP: Prob(AAP) = (eLP1/1 + eLP1) × (1 – eLP2 / 1 + eLP2).
- Probability of AIP: Prob(AIP) = (eLP1/1 + eLP1) × (eLP2/1 + eLP2).

Based on quantifiable US markers, this risk estimation method was crucial in our study, allowing for a nuanced and calculable assessment of the likelihood of various PAS categories.

### 2.2 Statistical analyses

Data were analyzed using R software version 4.0.4 (February 2021, R Foundation for Statistical Computing, Vienna, Austria). The study's aim was to validate the existing model proposed by Sargent et al. The authors decide to use the same statistical method as in the mentioned study. For the external validation, the model discrimination was assessed using the *C*‐Index, whereby values below 0.5 indicate a very poor model, 0.5 indicates a model no better at predicting an outcome than random chance, values over 0.7 indicate a good model, those over 0.8 indicate a strong model, and 1 indicates perfect prediction. *C*‐index is a widely accepted measure for binary classification problems. It is an excellent indicator of the level of agreement between predicted probabilities and actual outcomes. Additionally, calibration was assessed through a plot using flexible calibration curves, and the calibration intercept and slope were reported.^12^ It provides a comprehensive view of how well the model's predictions align with the actual risk levels. The univariable relationships between the descriptors and the ordinal outcomes were shown graphically. Ordinal logistic regression models were used to quantify univariable relationships between 12 standardized US markers and the presence or absence of AAP or AIP. To understand the maximum achievable model performance in this dataset, we developed a new model based on the FetView 2.0 database using the same methods as Sargent et al.,^12^ after inclusion of all significant variables. The *C*‐index and calibration plot are well‐established methods to assess model performance, and the author's decision to use them strengthens the validity of their findings.

## 3 RESULTS

### 3.1 Validation of the Sargent Model

**FIGURE 1:**
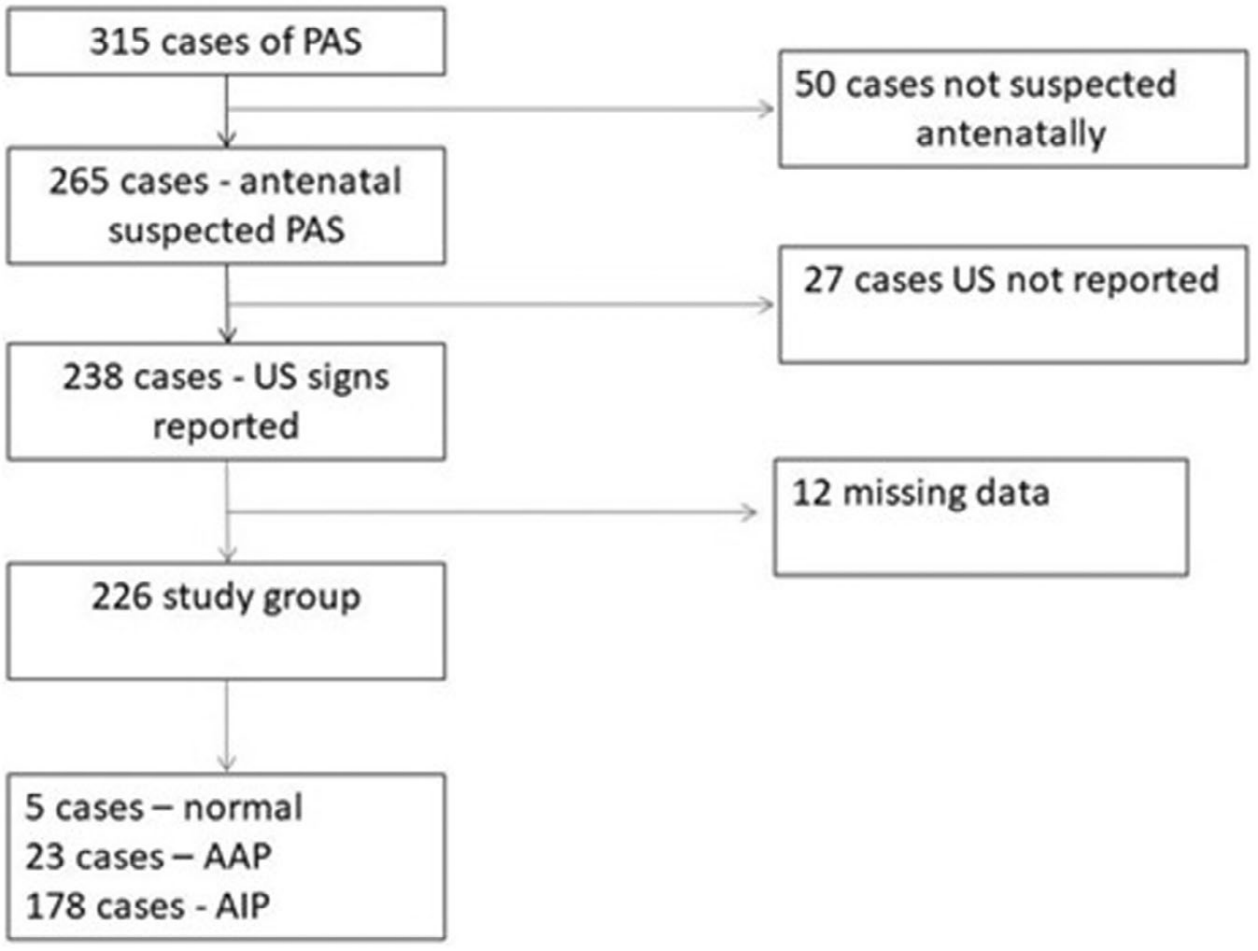
Flow chart with the final diagnosis of the study cohort.

The database included 315 cases of suspected PAS, of which 226 had fully documented standardized US signs. The final clinical and/or histopathological diagnosis was normal placentation in 5, AAP in 43, and AIP in 178. The flow chart of the database cases is presented in Figure 1.

**FIGURE 2:**
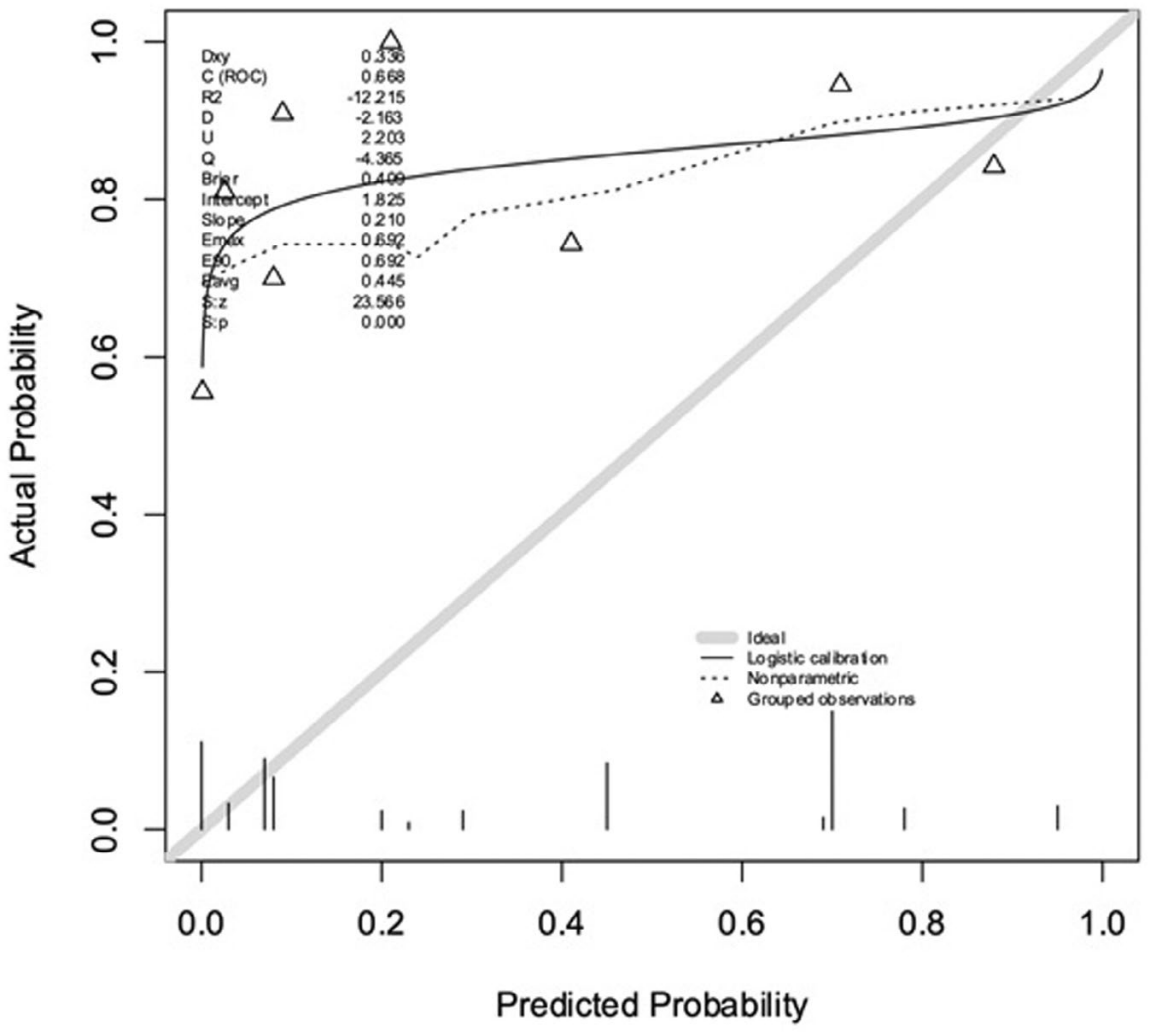
Probability prediction plot to differentiate between abnormally adherent placenta (AAP) and abnormally invasive placenta (AIP) using the prediction model of Sargent et al. A perfectly calibrated model would show a 45‐degree line (gray line). In this population, the model under‐predicts risk (black line).

The *C*‐index discrimination between AAP and AIP using the IS‐PAS database for the model proposed by Sargent et al. was 0.68 (approaching, but not reaching “good” prediction), and the calibration plot is shown in Figure 2. The plot shows suboptimal calibration in predicting the probability of PAS severity. A perfectly calibrated model would show a curve near the 45‐degree line, and therefore, in this population, the model substantially under‐predicts risk.

### 3.2 Development of the IS‐PAS Modified Sargent Model

**FIGURE 3:**
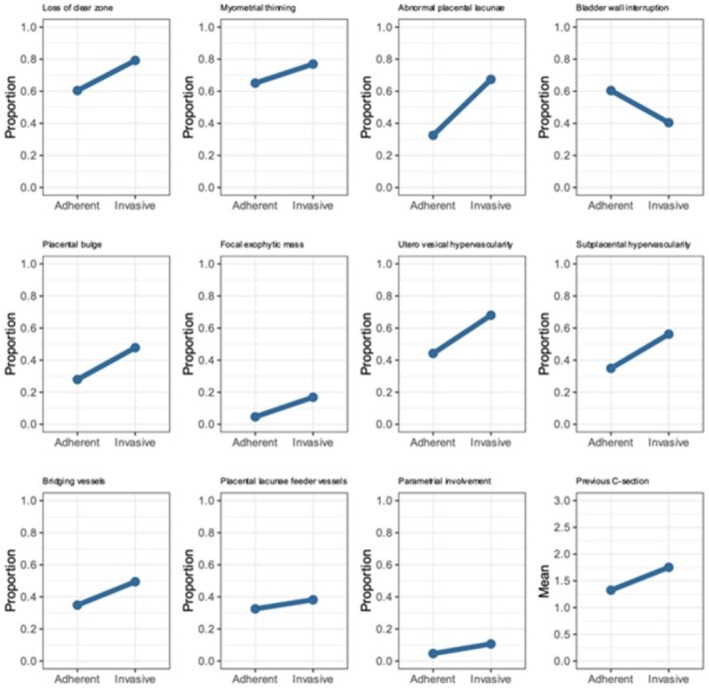
Plot of univariable relationships between the predictors and the outcome (adherent vs. invasive). *Y*‐axis showed the proportion of women with the US descriptors apart from previous cesarean delivery, which showed the mean number. All signs except bladder wall interruption were present in higher proportions in abnormally adherent placenta (AAP) versus abnormally invasive placenta (AIP). Most predictors showed a strong relationship, and abnormal placental lacunae were the strongest.

The univariable relationships between predictors and outcomes of the same 12 original US predictors originally analyzed by Sargent et al. were re‐analyzed using the data from IS‐PAS (FetView 2.0). Results were presented in plots (Figure 3). All signs except bladder wall interruption were present in higher proportions in AAP versus AIP. Most predictors showed a strong relationship, and abnormal placental lacunae were the strongest.

Univariable logistic regression analyses revealed six US features that significantly increased the risk of AIP compared to AAP. The most notable predictors included abnormal placental lacunae and focal exophytic mass, each increasing the odds fourfold, while loss of clear zone, placental bulge, uterovesical hypervascularity, and subplacental hypervascularity doubled the odds. Interestingly, the bladder wall interruption sign decreased the odds of AIP. These findings are detailed in Table 1 of the study.

**TABLE 1:** Results of the univariable logistic regression for the relationship of each investigated variable with the outcome (adherent vs. invasive).

Characteristic	*N*	OR	95% CI	*p*‐value
Loss of clear zone	221	2.49	1.21–5.06	**0.014**
Myometrial thinning	221	1.79	0.86–3.64	0.12
Abnormal placental lacunae	221	4.29	2.14–8.94	**<0.001**
Bladder wall interruption	221	0.44	0.22–0.87	**0.018**
Placental bulge	221	2.36	1.17–5.06	**0.017**
Focal exophytic mass	221	4.16	1.19–26.4	**0.023**
Uterovesical hypervascularity	221	2.68	1.36–5.34	**0.004**
Subplacental hypervascularity	221	2.39	1.21–4.89	**0.012**
Bridging vessels	221	1.83	0.92–3.72	0.083
Placental lacunae feeder vessels	221	1.28	0.64–2.66	0.49
Parametrial involvement	221	2.45	0.67–15.8	0.19
Previous C‐section	221	1.75	1.19–2.65	**0.004**

Abbreviations: CI, confidence interval; OR, odds ratio.

Statistically significant values are in bold.

Finally, in an exploratory analysis a new prediction model was created using the seven predictors identified using a stepwise logistic regression. This model achieved a *C*‐index of 0.74, indicating a fair level of predictive accuracy in a multicenter context but not a big improvement on Sargent et al. and also highlighting the challenges of developing a robust and universally applicable PAS prediction model.

## 4 DISCUSSION

Unlike all other published models, the Sargent model utilizes standardized US markers in line with the EW‐AIP (now IS‐PAS) consensus guidelines, providing a more replicable approach to PAS prediction.^13^ This model is designed to not only predict the presence of PAS but also differentiate between AAP and AIP.^12^

Our analysis revealed that while the Sargent model exhibited excellent predictive capability in a single‐center setting (*C*‐index of 0.90), its performance was less impressive when externally validated (*C*‐index of 0.68).^12^ This discrepancy underscores the challenges of developing universally applicable models in obstetrics. The calibration plot further highlighted this limitation, showing a tendency for the model to misclassify cases, particularly when attempting to distinguish between AAP and AIP. It might be related to a high proportion of AIP cases compared to AAP. However, other studies have also revealed similar observations that models developed within single centers often achieve lower accuracy when applied in other centers.^17^, ^18^, ^19^ For example, in 2021, Agarwal et al. evaluated the performance of Placenta Accreta Index in a prospective study of 45 patients. Their study highlighted moderate agreement among radiologists when applying the scale, reflecting the challenges in standardizing such predictive models across different centers.^17^ What is more, the Placenta Accreta Index is based on descriptors which are not used universally. Using the EW‐AIP and FIGO guidelines, myometrial thinning is described as <1 mm, whereas Rac et al.^6^ used 3 categories (<1 mm, 1–3 mm, 3–5 mm). Also, abnormal placental lacunae are usually classified as present when they correspond to Finberg grade 3 (not grades 2 and 3).^6^, ^13^, ^20^

A particularly surprising aspect of our study's findings was the poor performance of bladder wall interruption in PAS prediction. Counterintuitively, it appeared that bladder wall interruption was more commonly associated with decreased, rather than increased odds of deep extension in PAS. This observation challenges conventional wisdom and prompts reevaluation of how this US sign is defined and interpreted. Traditionally, bladder wall interruption was considered a significant indicator of deep extension. However, our study suggests that the current description of this sign may be too broad, potentially leading to misinterpretation. Specifically, when the bladder wall exhibits an equals sign (=) or scalloped edges, it is typically associated with abnormal neovascularity seen on the serosal surface in cases of AIP. This is a critical distinction, as the current definition does not accurately distinguish between actual pathological changes versus mere irregularities that might be a result of bladder wall reflection downward from previous cesarean deliveries. The reevaluation of this sign's effectiveness was echoed in the study by Philips et al.,^21^ which found that several markers commonly associated with PAS, including irregularity of the bladder wall, were often present in low‐risk pregnancies. This finding suggests that irregularities in the bladder wall, commonly observed in such cases, may not be as definitive of high‐risk PAS as previously assumed. To improve the diagnostic accuracy of this marker, it is crucial to refine its definition. This would involve distinguishing between US features indicative of serosal neovascularity—a sign of deep and complex PAS—and mere irregularities that could arise from previous surgical interventions such as cesarean delivery.

Attempts to improve the Sargent et al. model using the US data from the multicenter database, despite a *C*‐index of 0.74, which indicates a good model, were disappointing, as this was only an incremental improvement over the original Sargent's model. This may reflect the differences between PAS experts in interpretation of standardized descriptors, and highlights the need for the definitions to be revisited and refined in light of more recent developments in knowledge of the underlying anatomy.

It is important to emphasize the significance of training for the multidisciplinary team. Effective collaboration between the diagnostic and surgical groups is essential. This involves reporting imaging findings, receiving feedback, and auditing results, all of which contribute to enhancing diagnostic performance. The precise practices in each institution were not available in our database; however, it is possible that in single centers with robust full‐team training, accuracy may be more robust.

There are many published prognostic models. Tovbin et al. designed a scoring system for categorizing patients into low, medium, or high PAS risk groups.^8^ Yiso Gao's model incorporates maternal clinical history, such as parity, number of curettages, and cesarean sections, into the US‐based risk assessment.^9^ Fratelli et al. conducted the multicenter ADoPAD study, which underscored the effectiveness of grayscale US in identifying low‐risk PAS in high‐risk pregnancies.^10^ A summary of these studies and their findings is presented in Table S1. These diverse calculators highlight the evolving landscape of PAS prediction models, each contributing unique perspectives and methodologies to the field.

It is crucial to understand that constantly developing novel models at individual centers is not significantly improving the diagnosis of PAS on a global level. It is worth emphasizing that the risk of PAS is lowered by bladder wall interruption, and therefore, the marker needs to be reevaluated. To enhance the model's accuracy, it is worth considering including relevant medical history information.

The Sargent model has potential for clinical practice but may be challenging in low‐ and middle‐income countries due to limited access to imaging. With its user‐friendly diagram format, the Sargent model is a powerful tool in centers dedicated to PAS. The researchers believe that providing a workshop may generalize the topic of PAS and creating a mobile application may facilitate applicability.

The diagnosis of PAS in the future will involve assessing the effectiveness of machine learning and artificial intelligence.

The strength of the study was the use of the multicenter, prospectively collected US data. This enhances the generalizability and applicability of our findings across clinical settings. One of the key limitations was the relatively small number of normal placentation cases identified in the study. This prevented exploration of other prediction models which only provide a binary outcome of PAS or not PAS. Alternatively, it is possible that use of prediction models such as those we have evaluated are more robust in discerning presence versus absence of PAS than antenatal prediction of PAS severity. It is essential to keep potential biases in mind while interpreting the study's results. While selection bias had minimal impact since the model was not intended for use in the general population, it was validated in a group suspected of PAS and should only be used in such a population. Moreover, it is important to understand that all PAS signs may be present in the general population, and patients should be selected carefully. Therefore, it is crucial to apply the model only in the intended population. Additionally, observer bias in US interpretation may have slightly affected the study. However, a standardized protocol to perform US was implemented throughout all the centers to minimize the impact.

## 5 CONCLUSION

The study's findings highlight the difficulties inherent in developing a model to predict PAS. While promising, when using the data collected by a single operator in a single center, the Sargent model demonstrated less predictive accuracy when applied to data collected in other centers. The modified IS‐PAS model developed using similar methods to the Sargent's model gave a good level of predictive accuracy of PAS severity, but was still lower than that found in the original study.

Future research efforts should focus on updating the standardized US signs in light of an increased understanding of the underlying anatomical changes in PAS, and the development of newer PAS prediction models must be based on real‐world use of the refined definitions across several centers.

## AUTHOR CONTRIBUTIONS

Magdalena Kolak: writing—original draft, methodology, writing—review and editing. Stephen Gerry: Karin A. Fox Formal analysis, writing—review and editing. Hubert Huras, Zdenek Lastuvka, Pavel Calda and Egle Savukyne: Writing—review and editing. Ammar Al Naimi: Conceptualization, investigation, writing—review and editing. Thorsten Braun: Data curation, writing—review and editing. Vedran Stefanovic: Investigation, writing—review and editing. Heleen van Beekhuizen: Validation, writing—review and editing. Olivier Morel: Project administration, resources, writing—review and editing. Alexander Paping: Software, writing—review and editing. Charline Bertholdt: Methodology, writing—review and editing. Andrzej Jaworowski Writing—original draft, visualization, writing—review and editing. Sally Collins: Supervision, writing—review and editing, methodology. All IS‐PAS Group collaborators contributed by filling the forms with own cases, giving suggestions during preparation of manuscript and approved the final version.

## FUNDING INFORMATION

Karin A Fox receives funding from the Eunice Kennedy Shriver NICHD R01HD094347‐05. Pavel Calda is supported by the Ministry of Health, Czech Republic—conceptual development of research organization, General University Hospital in Prague, MH CZ—DRO‐VFN64165.

## CONFLICT OF INTEREST STATEMENT

All other authors have no conflicts of interest to declare.

## ETHICS STATEMENT

All centers have local ethical approval to submit anonymous data for the purposes of research to the database. All participating centers were responsible for contributing to clinical and scientific research under local IRB/ethics committee approval and operated under Data Use Agreements between individual centers and the IS‐PAS. Details of these have been published previously.^22^

## Supporting information


Table S1.

## APPENDIX A

### A.1

International Society for Placenta Accreta Spectrum (IS‐PAS) Group collaborators:

Kinga M. Chalubinski^13^, Katarzyna Kawka‐Paciorkowska^14^, Mina Mhallem Gziri^15^, Maddalena Morlando^16,17^, Frederic Chantraine^18^, Gita Strindfors^19^, Anja Bluth^20^, Petra Hanulikova^21^, Ozhan M Turan^22^, Andrew Rubenstein^23^, Albaro Jose Nieto‐Calvache^24,25^

^13^Department of Obstetrics and Gynecology, Division of Obstetrics and Feto‐Maternal Medicine, Medical University of Vienna, Vienna, Austria; ^14^Department of Perinatology and Gynecology, Poznan University of Medical Sciences, Poland; ^15^Department of Obstetrics, Cliniques Universitaires Saint‐Luc, Brussels, Belgium; ^16^Department of Woman, Child and General and Specialized Surgery, Obstetrics and Gynecology Unit, University of Campania “Luigi Vanvitelli”, Naples, Italy; ^17^Department of Neuroscience, Reproductive Sciences and Dentistry, University of Naples Federico II, Naples, Italy; ^18^Department of Obstetrics and Gynecology, Centre Hospitalier Universitaire de Liège, CHR Citadelle, Liège, Belgium; ^19^Department of Obstetrics and Gynecology, South General Hospital, Stockholm, Sweden; ^20^Department of Gynecology and Obstetrics, University Hospital Carl Gustav Carus, Technische Universität Dresden, Dresden, Germany; ^21^Institute for the Care of Mother and Child, Third Faculty of Medicine, Charles University, Prague, Czech Republic; ^22^Division of Maternal‐Fetal Medicine, Department of Obstetrics, Gynecology and Reproductive Science, University of Maryland School of Medicine, Baltimore, MD, USA; ^23^Department of Obstetrics and Gynecology, Saint Joseph's Hospital and Medical Center, Creighton University School of Medicine, Phoenix, AZ, USA; ^24^Fundacion Valle de Lili, Clinica de Espectro de Acretismo Placentario, Cali, Colombia; ^25^Facultad de ciencias de la salud, Universidad ICESI, Cali, Colombia
