# Supplementary material for: External validation of and improvement upon a model for the prediction of placenta accreta spectrum severity using prospectively collected multicenter ultrasound data
Source: Acta Obstet Gynecol Scand. 2024 Aug 20;104(Suppl 1):20–8. doi: 10.1111/aogs.14941 (PMC12087402; doi:10.1111/aogs.14941)
Supplement: Supplementary file 1 — Table S1. [file AOGS-104-20-s001.docx]

| **Author** | **Type** | **Cases (control/PAS)** | **% PAS** | **Variable** | **Description** | **Conclusion** |
| --- | --- | --- | --- | --- | --- | --- |
| Sargent et al.  2022 (12) | prospective | 106 (42/64) | 60.4 | Standardized US signs:  loss of clear zone, abnormal placenta lacunae, bladder wall interruption, placental bulge | Patients with previous CS and placenta praevia/anterior low lying placenta | High accuracy of model  C-index of 0.901 |
| Pain et al.  2022 (5) | retrospective | 82  (no control)  accreta/increta (35%)  vs. percreta (65%) | - | US: intraplacental lacunae without hyperechoic halo, increase vascularization at the uterine serosa-bladder wall interface  MRI: heterogeneous placenta, dark intraplacental bands | Patients with suspected PAS to differentiate between: accreta/increta and percreta  Created normograms | AUC 0.841 |
| Rac et al.  2015 (6) | retrospective | 88 (59/29) | 33 | US: loss of clear zone, bladder wall interruption, smallest myometrial thickness, lacunae, bridging vessels  Clinical: number of previous CS | Patients with previous CS and placenta praevia/ low lying placenta  Placenta Accreta Index (PAI) were created – nine point scale | Estimation of PAI index 2-96% |
| Maymon et al.  2018 (7) | retrospective | 471 (430/41) | 8.7 | US: lacunae, loss of clear zone, placenta praevia | Patients with previous CS.  Idea based on previous work (Tovbin et al.) | Estimation of PAS 1.5-87% |
| Tovbin et al.  2016 (8) | retrospective | 258 (235/23) | 8.9 | US: placenta location, number and size of lacunae, loss of clear zone,  Doppler US: hypervascularity of placenta bladder/uteroplacental interface, blood flow in placenta lacunae  Clinical: number of previous CS | Patients with previous CS.  Divided into groups of low, moderate and high probability of PAS | High prediction of PAS in pregnancy at risk  ROC curve 0.94 (using number of lacunae and loss of clear zone) |
| Yisu Gao et al.  2021 (9) | retrospective | Training model:  398 (308/90)    Validation model:  45 (22/23) | 29.2      51.1 | US: number of lacunae, maximum diameter of lacunae, myometrial thinning<1mm, subplacental hypervascularity, bladder wall interruption, placental bulge, location of placenta, placenta praevia  Clinical: previous CS>=1, parity >=4, prior curettage>=2 | Training and validation model. | ROC curve: 0.925, sensitivity: 82.6%, specificity: 81.8%, PPV: 82.6%, NPV:81.8% |
| Fratelli et al.  2022 (10) | Prospective, multicenter | 473 (PAS: 99) | 20.9 | US signs:  loss of clear zone, abnormal placenta lacunae, bladder wall interruption, | Patients with placenta praevia or low lying placenta, during the 3rd trimester US were suspected of PAS when there were at least one of three US signs present. | All three markers: sensitivity 50%, specificity 98.4%, DOR 60.8, PPV 89.1%, NPV 88.2%  (good tool to identify low pregnancy risk) |

Abbreviations: MRI - magnetic resonance imaging, AUC - Area under the ROC Curve, ROC - receiver operating characteristic, PPV - positive predictive value, NPV - negative predictive value, DOR - Diagnostic odds ratio, PAS - placenta accreta spectrum, US-ultrasound, AAP- abnormally adherent placenta, AIP - abnormal invasive placenta, PAI - Placenta Accreta Index, CS - cesarean section
